# Triorganoindium Reagents in Rh-Catalyzed C–H Activation/C–C Cross-Coupling Reactions of 2-Arylpyridines

**DOI:** 10.3390/molecules23071582

**Published:** 2018-06-29

**Authors:** Ricardo Riveiros, Rubén Tato, José Pérez Sestelo, Luis A. Sarandeses

**Affiliations:** Centro de Investigaciones Científicas Avanzadas (CICA) and Departamento de Química, Universidade da Coruña, E-15071 A Coruña, Spain; rriveiros@udc.es (R.R.); rubentatorodriguez@hotmail.com (R.T.)

**Keywords:** C–H activation, cross-coupling reactions, rhodium-catalyzed, indium organometallics

## Abstract

The activation of C–H bonds through catalytic reactions using transition metals is an important challenge in organic chemistry in which the intermediates are related to those produced in the classical cross-coupling reactions. As part of our research program devoted to the development of metal-catalyzed reactions using indium organometallics, a protocol for the C–H activation and C–C coupling of 2-arylpyridines with triorganoindium reagents under Rh(I) catalysis is reported. Under the optimized conditions, we found that Me_3_In and Ar_3_In reagents reacted with 2-arylpyridines and related compounds in the presence of Rh(PPh_3_)_3_Cl, in PhCl/THF (9:1), at 120 °C for 48 h, to afford the *ortho*-coupling products in moderate to good yields. The nitrogen atom in the pyridine ring acts as a directing group to assist the functionalization at the *ortho* position of the aryl group forming a new C–C bond at this position.

## 1. Introduction

Since our discovery [1,2], the palladium catalyzed cross-coupling reaction of indium(III) organometallics has been established as a useful tool in organic synthesis [3,4]. In this reaction, triorganoindium reagents (R_3_In) can efficiently couple with a variety of organic electrophiles under palladium catalysis with high atom economy [5,6,7,8]. The process can be extended to other organoindium reagents [9,10,11,12,13] and transition metals in catalysis [14,15,16,17].

**Scheme 1:**
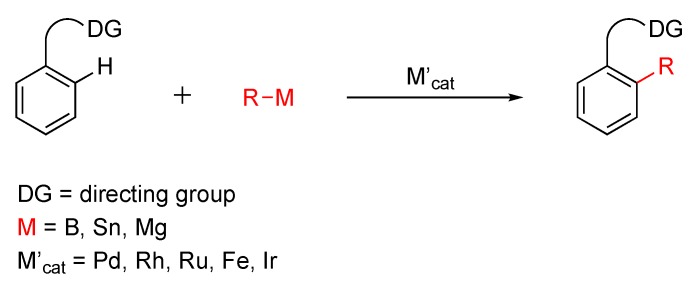
C–H activation/C–C cross coupling reactions.

The formation of C–C bonds by C–H activation and cross-coupling with organometallics and related nucleophiles under transition metal catalysis constitutes a methodology of great interest due to the atom economy of the process and the challenge that represent the selective functionalization of one C–H bond [18,19]. Usually, a heteroatom acts as directing group for the C–H activation through coordination with the metal center to form a metallacycle that reacts with the organometallic to afford the coupling product (Scheme 1) [20,21]. In this field, Oi et al. reported the Rh(I)-catalyzed *ortho*-arylation of 2-phenylpyridines with organotin compounds [22], a catalytic system also used in related reactions using boronic acids [23]. In 2003, Murai et al. reported the Ru(II)-catalyzed *ortho*-arylation of aryl ketones with boronates [24]. After these pioneering contributions, some coupling reactions of organometallic species with C–H bonds under different transition metal catalysis (Pd, Rh, Ru, Fe, Ir) have been reported [25,26,27,28,29,30]. Among them, the C–H activation of 2-phenylpyridines with organometallic reagents and related nucleophiles constitutes an interesting topic, developed under palladium [31,32] and iron catalysis [33]. Despite the novelty of this methodology, and to the best of our knowledge, organoindium compounds have not been applied as carbon nucleophiles in transition metal catalyzed C–H functionalization. Herein, we report the first examples of C–H activation/cross-coupling reaction of R_3_In with 2-arylpyridines and related compounds under Rh catalysis.

## 2. Results and Discussion

Our research started by testing the reaction of triorganoindium reagents with 2-(*p*-tolyl)pyridine (**1**) under Rh(I) catalysis as a model. In this reaction, the nitrogen atom should serve as a directing group to activate a C–H bond forming a metallacycle, which, by a transmetallation and reductive elimination process, should afford the C–H activation/C–C cross-coupling product. At the outset, we found that the reaction of Me_3_In (100 mol %) with **1** in the presence of Rh(PPh_3_)_3_Cl (10 mol %) at 120 °C in THF gave methylated compound **2a** in a low 16% yield after 48 h and most of the starting 2-(*p*-tolyl)pyridine recovered (Table 1, entry 1). Analogously, the reaction using Ph_3_In (100 mol %) gave **2b** in 17% yield along with important amounts of 1,1′-biphenyl, formed by reductive dimerization of the organoindium in the reaction media (entry 2). Using other Rh(I) complexes such as Rh(cod)(acac), Rh(nbd)(acac) or [Rh(cod)Cl]_2_ combined with phosphines did not improve the yields (entries 3–6). The influence of the solvent was also studied and, considering that R_3_In are prepared as THF solutions, various solvent mixtures were used. Using toluene/THF (9:1) the reaction with Ph_3_In (100 mol %) and Rh(PPh_3_)_3_Cl (10 mol %) at 120 °C for 48 h gave **2b** in a similar 18% yield (entry 7). Considering that polar non-protic chlorinated solvents have been successfully used in Rh(I)-catalyzed C–H activation reactions [22,34,35] 1,1,2,2-tetrachloroethane/THF (9:1) was tested as solvent, obtaining a 27% yield of **2b** and a 4% of the *o*,*o*′-disubstituted product **2bb** (entry 8). Considering that some of the starting 2-(*p*-tolyl)pyridine is recovered and part of the Ph_3_In is consumed with the formation of 1,1′-biphenyl, we decided to carry out the reaction using 150 mol % of Ph_3_In. Under these conditions, the yield of **2b** increased up to 42% (along with a 16% of **2bb**; entry 9). Analogously, the reaction using 150 mol % of Me_3_In also gave a satisfactory 60% yield of **2a** (entry 10). Finally, we found that using chlorobenzene as cosolvent the reaction results in a good 80% yield of **2a** using Me_3_In, and in 47% yield of **2b** in the reaction with Ph_3_In (entries 11 and 12). Recently, Li et al. reported the arylation of arylpyridines under rhodium catalysis using chlorobenzene as solvent [35]. Interestingly, we observed that the use of chlorobenzene diminishes the formation of the *o*,*o*′-disubstituted product and that the conversion of the starting material is not complete under the reaction conditions.

With the optimized conditions in hand, we explored the versatility of the reaction using various 2-arylpyridines. The reaction of Me_3_In with 2-phenylpyridine (**3**) yielded the methylated compound **7a** in a good 84% yield (and a 7% of the *o*,*o*′-dicoupled product **7aa**, Table 2, entry 3). Alternatively, the reaction of Ph_3_In with 2-phenylpyridine (**3**) afforded **7b** in 53% yield (6% of the disubstituted compound **7bb**, entry 4).

The reaction was also tested with 2-(2-naphthyl)pyridine (**4**), a compound with two nonsymmetrical *o*-positions of the aryl group. Under the optimized conditions, the reaction of **4** with Me_3_In and Ph_3_In allowed the synthesis of **8a** and **8b** in 60% and 57% yields, respectively (entries 5 and 6) without isolation of the double coupling products. The selectivity for the first C–H activation in a substrate where the two *ortho* positions are different in terms of electron density and steric hindrance is remarkable.

Additionally, we also tested the reactivity of 2-phenylpyrimidine (**5**), a substrate possessing two nitrogen atoms. The reaction with Me_3_In under the optimized conditions, afforded **9a** in 62% yield as the only product (entry 7). Using Ph_3_In, the monoarylated compound **9b** was obtained in 57% yield, accompanied by the formation of a 7% yield of the disubstituted product **9bb** (entry 8).

To improve the selectivity of the process, we used a rigid substrate such as benzo[*h*]quinoline (**6**) as starting material. In this case, the reaction of **6** with Me_3_In afforded **10a** as the only reaction product in an excellent 93% yield (entry 9). Furthermore, the reaction of **6** with Ph_3_In afforded compound **10b** in a 72% yield (entry 10). In these set of experiments, we demonstrated the efficiency of organoindium reagents in the transfer of methyl and phenyl groups in C–H coupling reactions with 2-arylpyridines.

At this point we also decided to test the versatility of the process using other triorganoindium reagents under the same reaction condition and using 1,1,2,2-tetrachloroethane as solvent. The reaction of trivinylindium with **6** afforded **10c** in a modest 25% yield (entry 11). Alternatively, the reaction of **6** with other triarylindium species such as tris[4-(trifluoromethyl)phenyl]indium, tris(4-fluorophenyl)indium and tris(3-fluorophenyl)indium, under the usual conditions, led to the compounds **10d**–**f** in moderated yields (20–45%; entries 12–14). Although the isolated yields of these last reactions might not be optimal, overall, these results represent the first examples of application of triorganoindium reagents in the Rh-catalyzed C–H activation and C–C cross-coupling with 2-arylpyridines and analogs and constitute a new entry in the reactivity of these compounds.

Although the mechanism of these reactions was not studied, based on previous reports [22,23,34], we believe the reaction proceeds via C–H activation of the *ortho* position of the 2-arylpyridine by an organorhodium(I) intermediate, which is generated by transmetallation of Rh(I)Cl species with the triorganoindium reagent. The resulting organoaryl(hydrido)rhodium species then undergoes reductive elimination to give the coupling product and a Rh(I)H species. Finally, the chlorinated solvent could participate in the regeneration of the active catalytic Rh(I) species by hydrogen/chloride exchange or HCl elimination. As for the proposal of Oi et al. [22], trichloroethylene was detected by ^1^H NMR of the reaction crude when 1,1,2,2-tetrachloroethane was used as solvent.

## 3. Materials and Methods

### 3.1. Materials and Reagents

All reactions were carried out in flamed dried glassware, under argon atmosphere, using standard gastight syringes, cannulas, and septa. Reaction temperatures refer to external bath temperatures. Anhydrous THF was obtained by distillation from the sodium ketyl of benzophenone. Chlorobenzene and 1,1,2,2-tetrachloroethane were dried and stored over microwave-activated 4 Å molecular sieves. All other commercially available reagents were used as received. Organolithium reagents (phenyllithium and methyllithium) were titrated prior to use. Organic extracts were dried over anhydrous MgSO_4_, filtered, and concentrated by using a rotary evaporator at aspirator pressure. Reactions were monitored by TLC using pre-coated silica gel plates (Alugram Xtra SIL G/UV_254_, 0.20 mm thick), UV light as the visualizing agent and ethanolic phosphomolybdic acid as the developing agent. Flash column chromatography was performed on silica gel (230–400 mesh). ^1^H and ^13^C NMR were recorded in CDCl_3_ at 300 MHz for ^1^H and 75 MHz for ^13^C, at 300 K, and calibrated to the solvent peak. DEPT data were used to assign carbon types (see Supplementary Materials). Mass spectra were obtained with EI ionization at 70 eV. IR spectra were recorded on a FT-IR spectrometer with an ATR (Attenuated Total Reflectance) accessory.

### 3.2. Preparation of Triorganoindium Reagents

Triorganoindium compounds were prepared according to previously published methods [36] by treatment of the corresponding organolithium reagent (3 equiv, ~0.5 M in THF) with a solution of InCl_3_ (1 equiv, 0.45 M in THF) at −78 °C and warming to room temperature. Organolithium reagents derived from 1-bromo-4-(trifluoromethyl)benzene, 1-bromo-4-fluorobenzene, 1-bromo-3-fluorobenzene and vinyl bromide were prepared by metal-halogen exchange reaction with *t*-BuLi (2 equiv) at −78 °C.

### 3.3. Preparation of Compounds ***4*** and ***5***

*2-(2-Naphthyl)pyridine* (**4**) [37]. To a solution of 2-bromopyridine (0.96 mL, 10.0 mmol) and Pd(dppf)Cl_2_ (408 mg, 0.5 mmol) in THF (50 mL), tri(2-naphthyl)indium (10.0 mmol, ~0.3 M in THF) was added. The mixture was stirred at 80 °C during 20 h, and the reaction was quenched by addition of drops of MeOH. The solvent was evaporated and EtOAc (25 mL) was added. The organic phase was washed with HCl (5%, 15 mL), satd. NH_4_Cl (15 mL) and brine (15 mL), dried, filtered, and concentrated. The crude was purified by flash chromatography (10% EtOAc/hexane), affording, after concentration and drying, **9** (1.05 g, 5.12 mmol, 51%) as a yellow solid. M.p. 69–70 °C (lit. [37], 77–78 °C); ^1^H NMR (CDCl_3_, 300 MHz) *δ* 7.27 (ddd, *J* = 7.2, 4.9, 1.0 Hz, 1 H), 7.50–7.55 (m, 2 H), 7.80 (td, *J* = 7.7, 1.8 Hz, 1 H), 7.87–7.97 (m, 4 H), 8.16 (dd, *J* = 8.6, 1.8 Hz, 1 H), 8.50 (d, *J* = 1.5 Hz, 1 H), 8.77 (ddd, *J* = 4.9, 1.5, 1.0 Hz, 1 H) ppm; ^13^C NMR (CDCl_3_, 75 MHz) *δ* 120.8 (CH), 122.1 (CH), 124.5 (CH), 126.3 (CH), 126.3 (CH), 126.5 (CH), 127.6 (CH), 128.4 (CH), 128.7 (CH), 133.5 (C), 133.6 (C), 136.6 (C), 136.8 (CH), 149.7 (CH), 157.3 (C) ppm; IR (ATR) 3053, 3010, 2919, 2849 cm^−1^; MS (EI) *m/z* (%) 205 (M^+^, 100), 204 ([M − H]^+^, 75); HRMS (EI) *m/z* calcd for C_15_H_11_N 205.0886, found 205.0881.

*2-Phenylpyrimidine* (**5**) [38]. To a solution of 2-chloropyrimidine (1.21 g, 10.0 mmol) and Pd(dppf)Cl_2_ (408 mg, 0.5 mmol) in THF (50 mL), triphenylindium (4.0 mmol, ~0.3 M en THF) was added. The mixture was stirred at 80 °C for 4 h and the reaction quenched by addition of drops of MeOH. The solvent was evaporated and EtOAc (25 mL) was added. The organic phase was washed with HCl (5%, 15 mL), satd. NH_4_Cl (15 mL) and brine (15 mL), dried, filtered, and concentrated. The crude was purified by flash chromatography (20% EtOAc/hexane) affording, after concentration and drying, **12** (1.42 g, 9.09 mmol, 91%) as a white solid. M.p. 36–38 °C (lit. [38], 36–38 °C). ^1^H NMR (CDCl_3_, 300 MHz) *δ* 6.95 (t, *J* = 4.9 Hz, 1 H), 7.42–7.49 (m, 3 H), 8.47–8.50 (m, 2 H), 8.66 (d, *J* = 4.9 Hz, 2 H) ppm; ^13^C NMR (CDCl_3_, 75 MHz) *δ* 119.0 (CH), 128.2 (2 × CH), 128.6 (2 × CH), 130.8 (CH), 137.5 (C), 157.1 (2 × CH), 164.5 (C) ppm; IR (ATR) 3087, 3066, 3039 cm^−1^; MS (EI) *m/z* (%) 156 (M^+^, 92), 103 (100); HRMS (EI) *m/z* calcd for C_10_H_8_N_2_ 156.0682, found 156.0683.

### 3.4. General Procedure C–H Activation/C–C Cross-Coupling with Indium(III) Organometallics under Rhodium Catalysis

In a Schlenk tube, a solution of R_3_In (0.375 mmol, ~0.3 M en THF) and the arylpyridine (0.25 mmol) were successively added to a solution of Rh(PPh_3_)_3_Cl (24 mg, 0.025 mmol) in chlorobenzene (20 mL). The mixture was stirred at 120 °C during 48 h, and the reaction quenched by addition of drops of MeOH. The solvent was evaporated and CHCl_3_ (25 mL) was added. The organic phase was washed with aq. NH_3_ (5%, 15 mL), dried, filtered, and concentrated. The crude was purified by flash chromatography (Et_2_O/hexane) affording, after concentration and drying, the cross-coupling products.

*2-(2,4-Dimethylphenyl)pyridine* (**2a**) [39]. Colorless oil (37 mg, 0.200 mmol, 80%). ^1^H NMR (CDCl_3_, 300 MHz) *δ* 2.35 (s, 3 H), 2.38 (s, 3 H), 7.10 (d, *J* = 7.7 Hz, 2 H), 7.23 (ddd, *J* = 7.5, 4.9, 1.0 Hz, 1 H), 7.31 (d, *J* = 7.5 Hz, 1 H), 7.39 (dt, *J* = 7.9, 1.0 Hz, 1 H), 7.73 (td, *J* = 7.7, 1.8 Hz, 1 H), 8.69 (ddd, *J* = 4.9, 1.8, 1.0 Hz, 1 H) ppm; ^13^C NMR (CDCl_3_, 75 MHz) *δ* 20.2 (CH_3_), 21.1 (CH_3_), 121.3 (CH), 124.0 (CH), 126.5 (CH), 129.6 (CH), 131.4 (CH), 135.5 (C), 135.9 (CH), 137.6 (C), 137.9 (C), 149.1 (CH), 160.1 (C) ppm; IR (ATR) 2954, 2922, 2853, 2360 cm^−1^; MS (EI) *m/z* (%) 183 (M^+^, 44), 182 ([M − H]^+^, 100); HRMS (EI) *m/z* calcd for C_13_H_13_N 183.1043, found 183.1027.

*2-(2,4,6-trimethylphenyl)pyridine* (**2aa**) [40]. Colorless oil (6 mg, 0.030 mmol, 12%) ^1^H NMR (CDCl_3_, 300 MHz) *δ* 2.03 (s, 6 H), 2.33 (s, 3 H), 6.94 (s, 2 H), 7.24–7.30 (m, 2 H), 7.18 (td, *J* = 7.7, 1.6 Hz, 1 H), 8.72–8.74 (m, 1 H) ppm; ^13^C NMR (CDCl_3_, 75 MHz) *δ* 20.0 (2 × CH_3_), 21.0 (CH_3_), 121.6 (CH), 124.8 (CH), 128.3 (2 × CH), 135.2 (C), 135.6 (2 × C), 136.5 (CH), 137.5 (C), 149.2 (CH), 159.7 (C) ppm; IR (ATR) 2952, 2921, 2855, 2358 cm^−1^; MS (EI) *m/z* (%) 197 (M^+^, 34), 196 ([M − H]^+^, 100); HRMS (EI) *m/z* calcd for C_14_H_15_N 197.1199, found 197.1181.

*2-(5-Methyl[1,1′-biphenyl]-2-yl)pyridine* (**2b**) [41]. White solid (29 mg, 0.118 mmol, 47%). ^1^H NMR (CDCl_3_, 300 MHz) *δ* 2.46 (s, 3 H), 6.86 (dt, *J* = 7.9, 1.0 Hz, 1 H), 7.08 (ddd, *J* = 7.5, 4.9, 1.0 Hz, 1 H), 7.14–7.19 (m, 2 H), 7.21–7.31 (m, 5 H), 7.36 (td, *J* = 7.7, 1.8 Hz, 1 H), 7.63 (d, *J* = 7.7 Hz, 1 H), 8.63 (ddd, *J* = 4.9, 1.8, 1.0 Hz, 1 H) ppm; ^13^C NMR (CDCl_3_, 75 MHz) *δ* 21.2 (CH_3_), 121.0 (CH), 125.3 (CH), 126.6 (CH), 128.0 (2 × CH), 128.3 (CH), 129.6 (2 × CH), 130.4 (CH), 131.2 (CH), 135.0 (CH), 136.7 (C), 138.3 (C), 140.4 (C), 141.5 (C), 149.3 (CH), 159.2 (C) ppm; IR (ATR) 3025, 2921, 2852 cm^−1^; MS (EI) *m/z* (%) 245 (M^+^, 32), 244 ([M − H]^+^, 100); HRMS (ESI) *m/z* calcd for C_18_H_16_N ([M + H]^+^) 246.1277, found 246.1275. 

*2-(5′-Methyl[1,1′:3′,1′’-terphenyl]-2′-yl)pyridine* (**2bb**) [42]. White solid. (5 mg, 0.015 mmol, 6%) ^1^H NMR (CDCl_3_, 300 MHz) *δ* 2.49 (s, 3 H), 6.85–6.92 (m, 2 H), 7.09–7.18 (m, 10 H), 7.26–7.32 (m, 3 H), 8.31 (ddd, *J* = 4.9, 1.6, 1.0 Hz, 1 H) ppm; ^13^C NMR (CDCl_3_, 75 MHz) *δ* 21.2 (CH_3_), 120.6 (CH), 126.1 (2 × CH), 126.8 (CH), 127.5 (4 × CH), 129.6 (4 × CH), 130.1 (2 × CH), 134.7 (CH), 135.8 (C), 137.7 (C), 141.7 (2 × C), 141.7 (2 × C), 148.4 (CH), 159.0 (C) ppm; IR (ATR) 3054, 3026, 2955, 2922, 2852 cm^−1^; MS (EI) *m/z* (%) 321 (M^+^, 52), 320 ([M − H]^+^, 100); HRMS (ESI) *m/z* calcd for C_24_H_20_N ([M + H]^+^) 322.1590, found 322.1589.

*2-(2-Methylphenyl)pyridine* (**7a**) [43]. Colorless oil (36 mg, 0.210 mmol, 84%). ^1^H NMR (CDCl_3_, 300 MHz) *δ* 2.38 (s, 3 H), 7.23–7.32 (m, 4 H), 7.39–7.43 (m, 2 H), 7.75 (td, *J* = 7.7, 1.9 Hz, 1 H), 8.71 (ddd, *J* = 4.9, 1.9, 1.0 Hz, 1 H) ppm; ^13^C NMR (CDCl_3_, 75 MHz) *δ* 20.2 (CH_3_), 121.5 (CH), 124.0 (CH), 125.8 (CH), 128.2 (CH), 129.6 (CH), 130.7 (CH), 135.7 (C), 136.0 (CH), 140.4 (C), 149.2 (CH), 160.0 (C) ppm; IR (ATR) 3059, 2924, 2359 cm^−1^; MS (EI) *m/z* (%) 169 (M^+^, 34), 168 ([M − H]^++^, 100); HRMS (EI) *m/z* calcd for C_12_H_10_N ([M − H]^+^) 168.0808, found 168.0814. 

*2-(2,6-dimethylphenyl)pyridine* (**7aa**) [39]. Colorless oil (3 mg, 0.016 mmol, 7%) ^1^H NMR (CDCl_3_, 300 MHz) *δ* 2.05 (s, 6 H), 7.10–7.12 (m, 2 H), 7.18–7.29 (m, 3 H), 7.77 (td, *J* = 7.7, 1.9 Hz, 1 H), 8.73 (ddd, *J* = 4.9, 1.9, 1.0 Hz, 1 H) ppm; ^13^C NMR (CDCl_3_, 75 MHz) *δ* 20.1 (2 × CH_3_), 121.6 (CH), 124.4 (CH), 127.5 (2 × CH), 127.8 (CH), 135.7 (2 × C), 136.2 (CH), 140.4 (C), 149.7 (CH), 159.9 (C) ppm; IR (ATR) 2924, 2854, 2360, 2214 cm^−1^; MS (EI) *m/z* (%) 183 (M^+^, 35), 182 ([M − H]^+^, 100); HRMS (EI) *m/z* calcd for C_13_H_12_N ([M − H]^+^) 182.0964, found 182.0963.

*2-[1,1′-Biphenyl]-2-yl-pyridine* (**7b**) [44]. White solid (31 mg, 0.132 mmol, 53%). M.p. 86–87 °C (lit. [44], 86.5–87.5 °C); ^1^H NMR (CDCl_3_, 300 MHz) *δ* 6.89 (dt, *J* = 7.9, 1.0 Hz, 1 H), 7.10 (ddd, *J* = 7.5, 4.9, 1.0 Hz, 1 H), 7.15–7.19 (m, 2 H), 7.22–7.25 (m, 3 H), 7.39 (td, *J* = 7.7, 1.8 Hz, 1 H), 7.41–7.51 (m, 3 H), 7.69–7.74 (m, 1 H), 8.64 (ddd, *J* = 4.9, 1.8, 1.0 Hz, 1 H) ppm; ^13^C NMR (CDCl_3_, 75 MHz) *δ* 121.3 (CH), 125.3 (CH), 126.6 (CH), 127.6 (CH), 128.0 (2 × CH), 128.5 (CH), 129.7 (2 × CH), 130.4 (2 × CH), 135.1 (CH), 139.4 (C), 140.6 (C), 141.3 (C), 149.4 (CH), 159.2 (C) ppm; IR (ATR) 3052, 3018, 2919, 2850 cm^−1^; MS (EI) *m/z* (%) 231 (M^+^, 26), 230 ([M − H]^+^, 100); HRMS (ESI) *m/z* calcd for C_17_H_14_N ([M + H]^+^) 232.1120, found 232.1123. 

*2-[1,1′:3′,1′’-terphenyl]-2′-yl-pyridine* (**7bb**) [44]. White solid (4 mg, 0.013 mmol, 6%) M.p. 123–124 °C (lit. [44], 118–119.5 °C); ^1^H NMR (CDCl_3_, 300 MHz) *δ* 6.88–6.94 (m, 2 H), 7.10–7.18 (m, 10 H), 7.30 (td, *J* = 7.7, 1.8 Hz, 1 H), 7.45–7.48 (m, 2 H), 7.52–7.57 (m, 1 H), 8.33 (ddd, *J* = 4.8, 1.8, 1.0 Hz, 1 H) ppm; ^13^C NMR (CDCl_3_, 75 MHz) *δ* 120.8 (CH), 126.2 (2 × CH), 126.7 (CH), 127.6 (4 × CH), 128.1 (CH), 129.4 (2 × CH), 129.6 (4 × CH), 134.8 (CH), 138.5 (C), 141.6 (2 × C), 141.8 (2 × C), 148.5 (CH), 158.9 (C); IR (ATR) 3056, 2923 cm^−1^; MS (EI) *m/z* (%) 307 (M^+^, 51), 306 ([M − H]^+^, 100); HRMS (ESI) *m/z* calcd for C_23_H_18_N ([M + H]^+^) 308.1433, found 308.1429.

*2-(3-Methyl-2-naphthyl)pyridine* (**8a**) [39]. White solid (33 mg, 0.150 mmol, 60%). M.p. 79–80 °C; ^1^H NMR (CDCl_3_, 300 MHz) *δ* 2.53 (s, 3 H), 7.29 (ddd, *J* = 8.2, 5.2, 1.5 Hz, 1 H), 7.42–7.53 (m, 3 H), 7.75–7.87 (m, 4 H), 7.89 (s, 1 H), 8.76 (ddd, *J* = 4.9, 1.8, 1.0 Hz, 1 H) ppm; ^13^C NMR (CDCl_3_, 75 MHz) *δ* 20.8 (CH_3_), 121.7 (CH), 124.2 (CH), 125.4 (CH), 126.3 (CH), 126.9 (CH), 127.9 (CH), 128.7 (CH), 128.8 (CH), 131.9 (C), 133.4 (C), 133.8 (C), 136.2 (CH), 139.4 (C), 149.2 (CH), 160.0 (C) ppm; IR (ATR) 3052, 3007, 2958, 2920, 2849 cm^−1^; MS (EI) *m/z* (%) 219 (M^+^, 57), 218 ([M − H]^+^, 100); HRMS (EI) *m/z* calcd for C_16_H_13_N 219.1043, found 219.1035.

*2-(3-Phenyl-2-naphthyl)pyridine* (**8b**) [44]. Brown solid (40 mg, 0.142 mmol, 57%). M.p. 107–108 °C (lit. [44], 103.5–106 °C); ^1^H NMR (CDCl_3_, 300 MHz) *δ* 6.98 (dt, *J* = 7.9, 1.0 Hz, 1 H), 7.14 (ddd, *J* = 7.5, 4.9, 1.0 Hz, 1 H), 7.28 (s, 5 H), 7.43 (td, *J* = 7.7, 1.8 Hz, 1 H), 7.52–7.55 (m, 2 H), 7.89–7.97 (m, 3 H), 8.22 (s, 1 H), 8.68 (ddd, *J* = 4.9, 1.8, 1.0 Hz, 1 H) ppm; ^13^C NMR (CDCl_3_, 75 MHz) *δ* 121.4 (CH), 125.5 (CH), 126.3 (CH), 126.7 (CH), 126.7 (CH), 127.7 (CH), 128.0 (2 × CH), 128.2 (CH), 129.5 (CH), 129.8 (2 × CH), 130.1 (CH), 132.6 (C), 133.2 (C), 135.1 (CH), 137.8 (C), 138.5 (C), 141.3 (C), 149.4 (CH), 159.1 (C) ppm; IR (ATR) 3054, 2923, 2853 cm^−1^; MS (EI) *m/z* (%) 281 (M^+^, 47), 280 ([M − H]^+^, 100); HRMS (EI) *m/z* calcd for C_21_H_14_N ([M − H]^+^) 280.1121, found 280.1126.

*2-(2-Methylphenyl)pyrimidine* (**9a**) [45]. Pale yellow oil (26 mg, 0.155 mmol, 62%). ^1^H NMR (CDCl_3_, 300 MHz) *δ* 2.56 (s, 3 H), 7.22 (t, *J* = 4.9 Hz, 1 H), 7.27–7.36 (m, 3 H), 7.80–7.83 (m, 1 H), 8.85 (d, *J* = 4.9 Hz, 2 H) ppm; ^13^C NMR (CDCl_3_, 75 MHz) *δ* 20.9 (CH_3_), 118.4 (CH), 125.8 (CH), 129.4 (CH), 130.3 (CH), 131.2 (CH), 137.1 (C), 138.1 (C), 156.8 (2 × CH), 167.7 (C) ppm; IR (ATR) 3034, 2924, 2853 cm^−1^; MS (EI) *m/z* (%) 170 (M^+^, 73), 169 ([M − H]^+^, 100); HRMS (EI) *m/z* calcd for C_11_H_10_N_2_ 170.0838, found 170.0830.

*2-[1,1′-Biphenyl]-2-yl-pyrimidine* (**9b**) [46]. White solid (33 mg, 0.142 mmol, 57%). M.p. 100–102 °C (lit. [46], 102–103 °C); ^1^H NMR (CDCl_3_, 300 MHz) *δ* 7.10 (t, *J* = 4.9 Hz, 1 H), 7.14–7.18 (m, 2 H), 7.21–7.27 (m, 3 H), 7.46–7.56 (m, 3 H), 7.80–7.83 (m, 1 H), 8.64 (d, *J* = 4.9 Hz, 2 H) ppm; ^13^C NMR (CDCl_3_, 75 MHz) *δ* 118.4 (CH), 126.4 (CH), 127.3 (CH), 127.9 (2 × CH), 129.1 (2 × CH), 129.3 (CH), 130.4 (CH), 130.7 (CH), 138.2 (C), 141.4 (C), 141.6 (C), 156.7 (2 × CH), 168.1 (C) ppm; IR (ATR) 3057, 3024, 2922, 2852, 2326 cm^−1^; MS (EI) *m/z* (%) 232 (M^+^, 25), 231 ([M − H]^+^, 100); HRMS (EI) *m/z* calcd for C_16_H_11_N_2_ ([M − H]^+^) 231.0917, found 231.0915. 

*2-[1,1′:3′,1′’-terphenyl]-2′-yl-pyrimidine* (**9bb**) [46]. White solid (5 mg, 0.016 mmol, 7%) M.p. 145–146 °C (lit. [46], 146–148 °C); ^1^H NMR (CDCl_3_, 300 MHz) *δ* 6.92 (t, *J* = 4.9 Hz, 1 H), 7.12–7.20 (m, 10 H), 7.48 (dd, *J* = 7.5, 1.0 Hz, 2 H), 7.57 (dd, *J* = 8.7, 6.4 Hz, 1 H), 8.46 (d, *J* = 4.9 Hz, 2 H) ppm; ^13^C NMR (CDCl_3_, 75 MHz) *δ* 118.0 (CH), 126.4 (2 × CH), 127.8 (4 × CH), 128.6 (CH), 129.1 (4 × CH), 129.2 (2 × CH), 137.6 (C), 141.3 (2 × C), 141.4 (2 × C), 155.9 (2 × CH), 168.1 (C) ppm; IR (ATR) 3058, 2918, 2850, 2324 cm^−1^; MS (EI) *m/z* (%) 308 (M^+^, 76), 307 ([M − H]^+^, 100); HRMS (EI) *m/z* calcd for C_22_H_15_N_2_ ([M − H]^+^) 307.1230, found 307.1231.

*10-Methylbenzo[h]quinoline* (**10a**) [38]. White solid (45 mg, 0.232 mmol, 93%). M.p. 70–71 °C; ^1^H NMR (CDCl_3_, 300 MHz) *δ* 3.40 (s, 3 H), 7.49 (dd, *J* = 8.0, 4.3 Hz, 1 H), 7.57–7.60 (m, 2 H), 7.66 (d, *J* = 8.8 Hz, 1 H), 7.80–7.83 (m, 2 H), 8.16 (dd, *J* = 8.0, 1.9 Hz, 1 H), 9.06 (dd, *J* = 4.3, 1.9 Hz, 1 H) ppm; ^13^C NMR (CDCl_3_, 75 MHz) *δ* 27.2 (CH_3_), 120.5 (CH), 125.4 (CH), 126.7 (CH), 127.2 (CH), 127.4 (C), 128.8 (CH), 129.9 (C), 131.1 (CH), 135.1 (C), 135.2 (CH), 138.7 (C), 147.1 (CH), 149.0 (C) ppm; IR (ATR) 3046, 2960, 2922, 2851, 2740 cm^−1^; MS (EI) *m/z* (%) 193 (M^+^, 100), 192 ([M − H]^+^, 66); HRMS (ESI) *m/z* calcd for C_14_H_12_N ([M + H]^+^) 194.0964, found 194.0961.

*10-Phenylbenzo[h]quinoline* (**10b**) [44]. Yellow oil (46 mg, 0.180 mmol, 72%). ^1^H NMR (CDCl_3_, 300 MHz) *δ* 7.33 (dd, *J* = 8.0, 4.3 Hz, 1 H), 7.36–7.42 (m, 5 H), 7.56 (dd, *J* = 7.3, 1.4 Hz, 1 H), 7.67–7.72 (m, 2 H), 7.87 (d, *J* = 8.8 Hz, 1 H), 7.94 (dd, *J* = 8.0, 1.4 Hz, 1 H), 8.09 (dd, *J* = 8.0, 1.9 Hz, 1 H), 8.44 (dd, *J* = 4.3, 1.9 Hz, 1 H) ppm; ^13^C NMR (CDCl_3_, 75 MHz) *δ* 121.0 (CH), 125.6 (CH), 125.9 (CH), 127.0 (CH), 127.2 (C), 127.3 (2 × CH), 127.9 (CH), 128.2 (CH), 128.7 (2 × CH), 129.0 (C), 131.4 (CH), 134.9 (C), 135.1 (CH), 141.7 (C), 146.4 (C), 146.8 (C), 146.8 (CH) ppm; IR (ATR) 3049, 3028, 2923 cm^−1^; MS (EI) *m/z* (%) 255 (M^+^, 27), 254 ([M − H]^+^, 100); HRMS (ESI) *m/z* calcd for C_19_H_14_N ([M + H]^+^) 256.1120, found 256.1113.

*10-Ethenylbenzo[h]quinoline* (**10c**) [47]. Yellow oil (13 mg, 0.0625 mmol, 25%). ^1^H NMR (CDCl_3_, 300 MHz) *δ* 5.41 (dd, *J* = 10.8, 1.9 Hz, 1 H), 5.63 (dd, *J* = 17.3, 1.9 Hz, 1 H), 7.51 (dd, *J* = 8.0, 4.3 Hz, 1 H), 7.65–7.70 (m, 2 H), 7.80–7.84 (m, 2 H), 7.89 (dd, *J* = 8.0, 1.3 Hz, 1 H), 8.18 (dd, *J* = 8.0, 1.9 Hz, 1 H), 8.61 (dd, *J* = 17.3, 10.8 Hz, 1 H), 9.06 (dd, *J* = 4.3, 1.9 Hz, 1 H) ppm; ^13^C NMR (CDCl_3_, 75 MHz) *δ* 112.5 (CH_2_), 120.9 (CH), 125.6 (CH), 127.6 (CH), 128.3 (CH), 128.54 (CH), 128.55 (CH), 134.7 (2 × C), 135.6 (CH), 139.2 (2 × C), 142.4 (CH), 147.6 (CH), 148.1 (C) ppm; IR (ATR) 3047, 2923, 2852, 2359 cm^−1^; MS (EI) *m/z* (%) 205 (M^+^, 27), 204 ([M − H]^+^, 100); HRMS (EI) *m/z* calcd for C_15_H_10_N ([M − H]^+^) 204.0808, found 204.0807.

*10-[4-(Trifluoromethyl)phenyl]benzo[h]quinoline* (**10d**) [48]. Yellow oil (16 mg, 0.0495 mmol, 20%). ^1^H NMR (CDCl_3_, 300 MHz) *δ* 7.35 (dd, *J* = 8.0, 4.3 Hz, 1 H), 7.46 (d, *J* = 8.0 Hz, 2 H), 7.51 (dd, *J* = 7.3, 1.1 Hz, 1 H), 7.66 (d, *J* = 8.0 Hz, 2 H), 7.71 (t, *J* = 7.3 Hz, 1 H), 7.72 (d, *J* = 8.8 Hz, 1 H), 7.89 (d, *J* = 8.8 Hz, 1 H), 7.98 (dd, *J* = 8.0, 1.4 Hz, 1 H), 8.11 (dd, *J* = 8.0, 1.8 Hz, 1 H), 8.40 (dd, *J* = 4.3, 1.8 Hz, 1 H) ppm; ^13^C NMR (CDCl_3_, 75 MHz) *δ* 121.3 (CH), 124.3 (q, ^3^*J*_CF_ = 3.8 Hz, 2 × CH), 124.7 (q, ^1^*J*_CF_ = 271.7 Hz, CF_3_), 126.1 (CH), 127.1 (CH), 127.3 (C), 127.7 (q, ^2^*J*_CF_ = 31.7 Hz, C), 128.2 (CH), 128.5 (CH), 128.8 (C), 128.9 (2 × CH), 131.1 (CH), 134.9 (C), 135.3 (CH), 140.2 (C), 146.4 (C), 146.9 (CH), 150.2 (C) ppm; IR (ATR) 2928 cm^−1^; MS (EI) *m/z* (%) 323 (M^+^, 30), 322 ([M − H]^+^, 100); HRMS (ESI) *m/z* calcd for C_20_H_13_NF_3_ ([M + H]^+^) 324.0994, found 324.0993.

*10-[4-(Fluorophenyl)benzo[h]quinoline* (**10e**) [48]. Yellow oil (31 mg, 0.113 mmol, 45%). ^1^H NMR (CDCl_3_, 300 MHz) *δ* 7.07–7.13 (m, 2 H), 7.27–7.37 (m, 3 H), 7.53 (dd, *J* = 7.3, 1.4 Hz, 1 H), 7.69 (t, *J* = 7.6 Hz, 1 H), 7.71 (d, *J* = 9.1 Hz, 1 H), 7.87 (d, *J* = 8.8 Hz, 1 H), 7.94 (dd, *J* = 8.0, 1.4 Hz, 1 H), 8.10 (dd, *J* = 8.0, 1.9 Hz, 1 H), 8.46 (dd, *J* = 4.3, 1.9 Hz, 1 H) ppm; ^13^C NMR (CDCl_3_, 75 MHz) *δ* 114.1 (d, ^2^*J*_CF_ = 21.3 Hz, 2 × CH), 121.1 (CH), 126.0 (CH), 127.0 (CH), 127.2 (C), 128.1 (CH), 128.3 (CH), 129.0 (C), 130.1 (d, ^3^*J*_CF_ = 7.8 Hz, 2 × CH), 131.5 (CH), 135.0 (C), 135.3 (CH), 140.6 (C), 142.2 (d, ^4^*J*_CF_ = 3.5 Hz, C), 146.7 (C), 146.8 (CH), 161.5 (d, ^1^*J*_CF_ = 243.1 Hz, CF) ppm; IR (ATR) 3049, 2926, 2870 cm^−1^; MS (EI) *m/z* (%) 273 (M^+^, 28), 272 ([M − H]^+^, 100); HRMS (ESI) *m/z* calcd for C_19_H_13_NF ([M + H]^+^) 274.1026, found 274.1022.

*10-[3-(Fluorophenyl)benzo[h]quinoline* (**10f**). Yellow oil (21 mg, 0.075 mmol, 30%). ^1^H NMR (CDCl_3_, 300 MHz) *δ* 7.03–7.08 (m, 2 H), 7.12–7.15 (m, 1 H), 7.32–7.38 (m, 2 H), 7.54 (dd, *J* = 7.3, 1.4 Hz, 1 H), 7.70 (t, *J* = 7.6 Hz, 1 H), 7.72 (d, *J* = 8.8 Hz, 1 H), 7.87 (d, *J* = 8.8 Hz, 1 H), 7.96 (dd, *J* = 8.0, 1.4 Hz, 1 H), 8.11 (dd, *J* = 8.0, 1.9 Hz, 1 H), 8.46 (dd, *J* = 4.3, 1.9 Hz, 1 H) ppm; ^13^C NMR (CDCl_3_, 75 MHz) *δ* 112.4 (d, ^2^*J*_CF_ = 21.1 Hz, CH), 115.8 (d, ^2^*J*_CF_ = 21.5 Hz, CH), 121.2 (CH), 124.4 (d, ^4^*J*_CF_ = 2.7 Hz, CH), 126.1 (CH), 127.0 (CH), 127.2 (C), 128.2 (CH), 128.3 (CH), 128.7 (d, ^3^*J*_CF_ = 8.5 Hz, CH), 128.9 (C), 131.1 (CH), 134.9 (C), 135.2 (CH), 140.3 (d, ^4^*J*_CF_ = 2.0 Hz, C), 146.5 (C), 147.0 (CH), 148.6 (d, ^3^*J*_CF_ = 8.3 Hz, C), 162.4 (d, ^1^*J*_CF_ = 243.3 Hz, CF) ppm; IR (ATR) 3048, 2955, 2924, 2854 cm^−1^; MS (EI) *m/z* (%) 273 (M^+^, 30), 272 ([M − H]^+^, 100); HRMS (ESI) *m/z* calcd for C_19_H_13_NF ([M + H]^+^) 274.1026, found 274.1023.

## 4. Conclusions

In summary, we report a novel protocol for the C–H activation and C–C coupling of 2-arylpyridines with triorganoindium reagents under rhodium catalysis. Reactions proceeded regioselectively to the *ortho* position of the aryl group with methyl- and phenylindium reagents in moderate to good yields. The use of chlorobenzene as solvent is useful to minimize the *o*,*o*′-disubstituted products. This contribution represents a new entry about the reactivity of organoindium reagents and could help to develop further studies on C–H activation reactions. Additionally, the reactions reported represent a new example for the transmetallation of organic groups from indium to rhodium.

## Figures and Tables

**Table 1:**
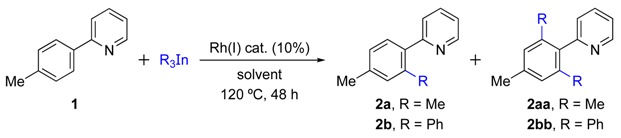
Reaction of indium organometallics with 2-(*p*-tolyl)pyridine under rhodium catalysis ^1^.

Entry	R (mol % of R_3_In)	Catalyst	Solvent	Yield (%) ^2^
1	Me (100)	Rh(PPh_3_)_3_Cl	THF	16
2	Ph (100)	Rh(PPh_3_)_3_Cl	THF	17
3	Me (100)	Rh(cod)(acac)	THF	14
4	Me (100)	Rh(nbd)(acac)	THF	15
5 ^3^	Me (100)	[Rh(cod)Cl]_2_, Ph_3_P	THF	14
6 ^3^	Me (100)	[Rh(cod)Cl]_2_, Cy_3_P	THF	15
7	Ph (100)	Rh(PPh_3_)_3_Cl	Toluene/THF (9:1)	18
8	Ph (100)	Rh(PPh_3_)_3_Cl	Cl_2_CHCHCl_2_/THF (9:1)	27 (4)
9	Ph (150)	Rh(PPh_3_)_3_Cl	Cl_2_CHCHCl_2_/THF (9:1)	42 (16)
10	Me (150)	Rh(PPh_3_)_3_Cl	Cl_2_CHCHCl_2_/THF (9:1)	60 (34)
11	Me (150)	Rh(PPh_3_)_3_Cl	PhCl/THF (9:1)	80 (12)
12	Ph (150)	Rh(PPh_3_)_3_Cl	PhCl/THF (9:1)	47 (6)

^1^ Reactions performed in a Schlenk tube using 10 mol % of the rhodium complex at 120 °C for 48 h. ^2^ In parentheses, yield of the *o*,*o*′-dicoupling product (**2aa**, **2bb**) obtained. ^3^ 5 mol % of rhodium complex and 20 mol % of phosphine used.

**Table 2:**
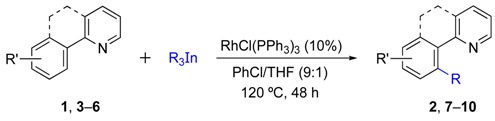
Reaction of R_3_In with 2-arylpyridines under rhodium catalysis ^1^.

Entry	2-Arylpyridine	R	Product	Yield (%) ^2^
1	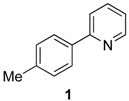	Me	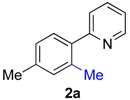	80 (12)
2		Ph	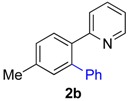	47 (6)
3	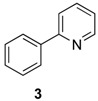	Me	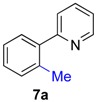	84 (7)
4		Ph	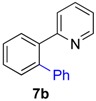	53 (6)
5	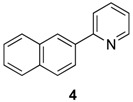	Me	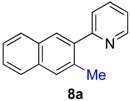	60
6		Ph	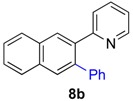	57
7	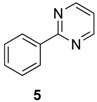	Me	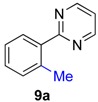	62
8		Ph	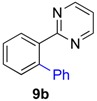	57 (7)
9	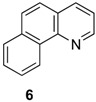	Me	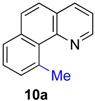	93
10		Ph	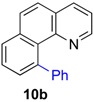	72
11		CH_2_=CH	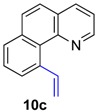	25 ^3^
12		4-CF_3_C_6_H_4_	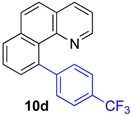	20 ^3^
13		4-FC_6_H_4_	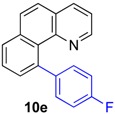	45 ^3^
14		3-FC_6_H_4_	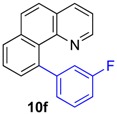	30 ^3^

^1^ Reactions performed in a Schlenk tube using 10 mol % of the rhodium complex at 120 °C for 48 h. ^2^ In parentheses, yield of the *o*,*o*′-dicoupling product obtained. ^3^ Cl_2_CHCHCl_2_/THF (9:1) was used as solvent.

## Supplementary Materials

The following are available online at http://www.mdpi.com/1420-3049/23/7/1582/s1: Copies of ^1^H NMR and ^13^C NMR spectra of the compounds **2**, **4**, **5** and **7**–**10**.
